# Genome-wide CRISPR screen reveals *PSMA6* to be an essential gene in pancreatic cancer cells

**DOI:** 10.1186/s12885-019-5455-1

**Published:** 2019-03-21

**Authors:** Jesse Bakke, William C. Wright, Anthony E. Zamora, Peter Oladimeji, Jeremy Chase Crawford, Christopher T. Brewer, Robert J. Autry, William E. Evans, Paul G. Thomas, Taosheng Chen

**Affiliations:** 1 Department of Chemical Biology and Therapeutics, St. Jude Children’s Research Hospital, Memphis, TN USA; 2 Department of Foundational Sciences, College of Medicine, Central Michigan University, Mount Pleasant, MI USA; 3 Integrated Biomedical Sciences Program, University of Tennessee Health Science Center, Memphis, TN USA; 4 Department of Immunology, St. Jude Children’s Research Hospital, Memphis, TN USA; 5 Department of Pharmaceutical Sciences, St. Jude Children’s Research Hospital, Memphis, TN USA

**Keywords:** CRISPR screen, PDAC, Pancreatic cancer, *PSMA6*, Essential genes

## Abstract

**Background:**

Despite its relatively low incidence, pancreatic ductal adenocarcinoma (PDAC) is a leading cause of cancer deaths because of the aggressive growth/metastasis of the tumor, the lack of early symptoms, and the poor treatment options. Basic research to identify potential therapeutic targets for PDAC is greatly needed.

**Methods:**

We used a negative-selection genome-wide CRISPR screen to identify essential genes in the PANC-1 human pancreatic carcinoma cell line. We validated the top hits with follow-up siRNA screens, using the HPNE, HPAF-II, AsPC-1, and Mia PaCa-2 cell lines.

**Results:**

The *PSMA6* gene was an identified candidate hit after the CRISPR screen, siRNA validation screen, and siRNA deconvolution screen. Spheroid formation assays and flow cytometry analysis showed that *PSMA6* is critical for survival in many pancreatic ductal carcinoma cell models. Lastly, as PSMA6 protein is a proteosomal subunit of the 20S core complex, we showed that bortezomib, a proteasome inhibitor, was especially toxic in PANC-1 cells.

**Conclusions:**

Further study of *PSMA6* and the proteasome subunit that it encodes, along with other hits identified in our CRISPR screens, may provide valuable insights into potential therapeutic targets for PDAC.

**Electronic supplementary material:**

The online version of this article (10.1186/s12885-019-5455-1) contains supplementary material, which is available to authorized users.

## Background

As of 2018, pancreatic cancer is the fourth leading cause of cancer-related deaths in the USA, with 55,000 new cases and 44,000 deaths reported annually. The mean 5-year survival of patients with pancreatic cancer is less than 8% [1]. Pancreatic ductal adenocarcinomas (PDACs) account for the vast majority of pancreatic cancer cases and are characterized by highly invasive mucin-producing neoplasms that commonly originate from noninvasive epithelial neoplasia of pancreatic ducts [2]. Through intensive research efforts, driver mutations have been identified in four genes: the oncogene *KRAS* and the tumor suppressors *CDKN2A*, *TP53*, and *SMAD4* [3]. Early mutations in *KRAS* and *CDKN2A* (which encodes the tumor suppressor protein P16) are present in more than 90% of all PDAC cases, whereas late mutations in *SMAD4* and *TP53* are present in approximately half of PDAC cases [4, 5]. Along with these driver mutations, recent large-scale sequencing and bioinformatic endeavors have implicated other biological processes, such as axon guidance, in the development of PDAC [6]. Despite the identification of driver mutations and the abundance of genomic data, it has proved difficult to identify novel therapeutically relevant targets, and this is reflected in the extremely poor prognosis of PDAC. More functional research efforts are required to identify therapeutic targets that may lead to new agents to improve the treatment and outcomes of PDAC.

To identify novel therapeutic targets of PDAC, we leveraged a genome-wide CRISPR screening approach that allowed us to quantify gene-specific phenotypic variation in PANC-1 cells in response to gemcitabine, the most commonly used PDAC chemotherapeutic. Genome-wide CRISPR screens are pool-based screening strategies that leverage the unique gRNA sequences and next-generation sequencing (NGS) to identify shifts in gRNA frequency after a phenotypic selection event [7, 8]. These screens are extremely robust [9] and have been used to identify genes that are essential for cell survival [10], that are involved in oxidative phosphorylation [11], and that confer drug resistance [12], among other important biological pathways. Gemcitabine is one of the most widely used chemotherapeutics for all stages of PDAC, despite its suboptimal efficacy and the rapid development of chemotherapy resistance. By using the genome-wide CRISPR screening approach, we aimed to identify genes that were essential to the survival of PANC-1 cells (our PDAC model of choice) and/or genes that sensitized PANC-1 cells to low-dose gemcitabine treatment. We then compared the regulatory effects of the identified genes on the survival of PANC-1 cells to their effects in a noncancerous pancreatic cell model, hTert-HPNE cells, and in other PDAC cell lines (AsPC-1, Mia PaCa-2, and HPAF-II) in an effort to identify PDAC pan-essential genes that were not required in normal pancreatic cells.

We validated this screening pipeline for identifying genes essential to several cellular models of PDAC. To that end, we interrogated a top candidate gene, proteasome subunit alpha type-6 (*PSMA6*), and confirmed that it is uniquely essential in the PDAC cells tested, but not in the noncancerous HPNE pancreatic cells. We were unable to identify a gene that had a synergistic relation with gemcitabine in all PDAC models, likely because of the multitude of drug transporters involved and the pathways disturbed by gemcitabine [13, 14].

## Methods

### Materials

Fetal bovine serum was purchased from HyClone (Logan, UT). Cell culture reagents, fluorescent secondary antibodies, and RNAiMAX transfection reagent were purchased from Invitrogen (Carlsbad, CA). All siRNAs (custom cherry-picked libraries) were purchased from Dharmacon (Lafayette, CO). *PSMA6* and *18S* TaqMan probes were purchased from Thermo Fisher Scientific (Waltham, MA).

### Cell culture

All cell lines were maintained in a humidified incubator at 37 °C in 5% CO_2_. PANC-1, hTert HPNE, Mia PaCa-2, HPAF-II, and AsPC-1 cells were purchased from ATCC and used experimentally within five passages. All cell lines were maintained according to ATCC recommendations, and ATCC authenticated the cell lines by short tandem repeat (STR) DNA profiling. The cells were verified to be mycoplasma-free by using the MycoProbe Mycoplasma Detection kit (R&D Systems, Minneapolis, MN). Cas9 stable cell lines were made by virally transducing cells with LentiCAS9-Blast (Addgene, Cambridge, MA; cat. # 52962) [15] and selecting with 8 μg/mL of blasticidin for 5 days. Expression was verified by Western blot analysis (Additional file 1b).

### CRISPR screen

Stable Cas9-expressing PANC-1 cells were transduced with the CRISPR lentiviral library at an experimentally established MOI of 0.3 in the presence of 4 μg/mL of polybrene overnight. The cells were selected with 2 μg/mL of puromycin for 9 days, at which point 1 × 10^8^ cells were collected and frozen for genomic DNA isolation. A further 1 × 10^8^ cells were grown in the presence of 100 nM gemcitabine for 6 days, after which the cells were frozen for genomic DNA isolation. Sequencing was performed on the Illumina HiSeq 2500 platform (100 bp SE), and raw FASTQ files were deconvoluted by barcode and trimmed of excess nucleotides by using custom scripts on the St. Jude Children’s Research Hospital high-performance computing facility. The resulting amplicons were then analyzed with MAGeCK-VISPR [16].

### Genomic DNA isolation and PCR amplification

Genomic DNA was extracted with QIAamp Blood Maxi kit (Qiagen, cat. # 51192) in accordance with the manufacturer’s protocol. Using a nested PCR program, we generated barcoded amplicons containing the integrated gRNA sequences. Briefly, 10 separate 100-μL redundant reactions were performed, each containing 5 μg of DNA, Premix Ex Taq HS (TaKaRa, cat. # RR030A), and 6 μL of a 10 μM solution of each primer (F1 and R1) (Additional file 2). The first round of the PCR amplification program was as follows: step 1, 95 °C for 1 min; step 2, 95 °C for 30 s; step 3, 55 °C for 30 s; and step 4, 72 °C for 30 s; with steps 2–4 being repeated 15 times. Then, 5 μL of the PCR product was used to seed the second round of PCR, along with Premix Ex Taq HS, 6 μL of the R2 primer, and 6 μL of a 10 μM solution of the F2 primer in a staggered mixture that contained the Illumina adapters and a barcode to identify the sample after sequencing analysis (Additional file 2). The second-round PCR program was as follows: step 1, 95 °C for 1 min; step 2, 95 °C for 30 s; step 3, 63 °C for 30 s; and step 4, 72 °C for 30 s; with steps 2–4 being repeated 17 times.

### siRNA confirmation screens

Top CRISPR screen hits were validated and deconvoluted with siRNA (on-target) from Dharmacon. Briefly, siRNA (25 nM) was mixed with 0.09 μL of RNAiMAX and Opti-MEM (Thermo Fischer Scientific). To generate heat maps and movies, 2000 cells were added to each well and the plates were analyzed with an IncuCyte Live Cell Analysis System (Essen BioScience, Inc., Ann Arbor, MI) for 3–5 days (as indicated in the figures), with the confluence of the cells being tracked every 4 h. We used 1 μM staurosporine as a positive control for cytotoxicity, and lipid only and a non-targeting siRNA were used as negative controls, with the data being normalized to these controls. Heat maps were generated after data normalization by using GraphPad Prism (GraphPad Software, La Jolla, CA). siRNAs targeting *PSMA6* (sequences AGACUAAACAUUGUCGUUA, CCUCUUGGUUGUUGUAUGA, CUACAGAGGGCACGCUAUCG, and GGUUACUACUGUGGGUUUA) were purchased from Dharmacon (cat. #s J-011360-05, J-011360-06, J-011360-07, and J-011360-08).

### RNA extraction and quantitative reverse transcription PCR

RNA was extracted with a Maxwell RSC simplyRNA Tissue Kit and a Maxwell RSC Instrument (Promega, Madison, WI). The RNA concentration was measured with a NanoDrop 8000 UV-Vis Spectrophotometer (Thermo Fisher Scientific). A SuperScript VILO cDNA Synthesis Kit (Life Technologies, Carlsbad, CA) was used to synthesize cDNA according to the manufacturer’s protocol. To determine mRNA expression, Applied Biosystems TaqMan assays (20×), Fast Advanced Master Mix (Life Technologies), and an Applied Biosystems 7900HT Fast Real-Time PCR System (Life Technologies) were used in accordance with the TaqMan Fast protocol. Gene expression was normalized to the 18S rRNA housekeeping gene, which did not vary in its expression during the growth of the cell lines. Each experiment was performed at least three times, and all samples were analyzed in triplicate.

### Lentivirus generation and viral transduction

Lentivirus was generated in HEK293T cells (ATCC, Manassas, VA) in 225-cm^2^ flasks. Briefly, 22.2 μg of a CRISPR pooled gRNA library (human sgRNA library Brunello in lentiGuide-Puro) transfer vector (Addgene, cat. #73178) [17], 16.7 μg of psPAX2 plasmid (Addgene, cat. # 12260), and 11 μg of pMD2.G plasmid (Addgene, cat. # 12259) were combined with Lipofectamine 3000 (Thermo Fisher Scientific) in accordance with the manufacturer’s protocol, and the mixture was used to transfect the cells. The virus-containing medium was collected 48 h after transfection and centrifuged at 500×*g* for 5 min to remove cells and debris. The supernatant containing the virus was then filtered with a 0.45-μM PES filter and frozen at − 80 °C. Viral transduction was accomplished by adding 150 μL of virus-containing medium per 1 × 10^6^ cells (titer determined experimentally, MOI = 0.3) to 225-cm^2^ flasks of PANC-1 cells at 75% cellular confluence, along with 4 μg/mL polybrene (Sigma-Aldrich), for 16 h. The virus-containing medium was then replaced with fresh growth medium.

### Determination of titer

A series of ten-fold **s**erial dilutions of the lentivirus-containing supernatant was used to determine the MOI. Briefly, in six-well plates, serially diluted lentiviral supernatant (in replicates of six, one plate per concentration) was added, along with 4 μg/mL of polybrene, to 200,000 PANC-1 cells, and the plates were incubated overnight. Twenty-four hours after the transduction, 2 μg/mL of puromycin was added to half of the samples at a given lentiviral concentration. After 3 days, the cells were counted and the counts compared to those for non-puromycin controls. Infection rates were determined as the ratio of cells under puromycin selection to cells not under puromycin selection. Values were plotted, and the volume that corresponded to an infection rate of 30% was used (MOI = 0.3).

### Flow cytometry

To determine the stage of apoptotic cell death in control and treated PANC-1 and Mia PaCa-2 cells, we performed flow cytometric analysis on PANC-1 and Mia PaCa-2 cells grown in vitro, using the PE Annexin V Apoptosis Detection Kit I (BD Biosciences, San Jose, CA) in accordance with the manufacturer’s protocol. Briefly, cells were washed twice with cold PBS and resuspended in 1× Binding Buffer at a concentration of 2 × 10^6^ cells/mL. Aliquots of 200 μL of the solution (containing 4 × 10^5^ cells) were transferred to 5-mL round-bottom tubes, then 5 μL of PE Annexin V and 5 μL of 7-AAD cell viability dye were added to the tubes. The cells were gently vortexed and incubated for 15 min. at room temperature while protected from light. Next, 400 μL of 1× Binding Buffer was added to each tube and the samples were immediately analyzed on a custom-configured BD Fortessa cytometry analyzer using FACSDiva software (Becton-Dickinson, San Jose, CA). Data were analyzed using FlowJo software (TreeStar, Ashland, OR). All experiments were performed with at least three biological replicates, and at least 200,000 events were collected per sample.

### Stable cell line generation

Three individual pools of Tet-on shRNA stable PANC-1 cells were generated after lentiviral transduction of early passage PANC-1 cells with SMARTvector (hEF1a) inducible *PSMA6* shRNA plasmids (Dharmacon 1255-01EG5687shRNA sequences: TAGAGTCCTAACCACTTCG, GATCTGGAAACTAACGAC, ACAGGTAAGTGGCATCACG). PANC-1 cells were selected with puromycin (2 μg/ml) for 3 days then analyzed for knockdown efficiency and stored with Bambanker Serum Free Freezing Media (Wako Chemicals #302–14,681) in liquid nitrogen vapor phase for future studies. Knockdown efficiency was tested 3 days post doxycycline treatment and mRNA of *PSMA6* was compared to the same stable cell line not treated with doxycycline, ACAGGTAAGTGGCATCACG sequence had a > 80% knockdown of *PSMA6* (Fig. 4f) and was used for subsequent studies. All inducible stable cell lines were maintained in tetracycline screened fetal bovine serum (Hyclone, Logan UT).

### Western blot

Alpha tubulin and PSMA6 antibodies were purchased from Cell Signaling Technologies (Boston, MA). Briefly, PANC-1 cells were treated with 25 nM siControl (non-targeting) or siPSMA6 for 72 h, lysed with RIPA buffer, and supernatant was collected for gel electrophoresis. PVDF membrane was probed with alpha tubulin and PSMA6 antibodies and imaged on a Li-Cor FC and bands were analyzed with Li-Cor Odyssey software.

### 3D-spheroid formation assay

PANC-1 cells stably expressing shRNA targeting *PSMA6* were seeded into a round-bottom 96-well plate at a density of 300 cells/well. The medium was changed every 3–4 days, and spheroid images were captured using an IN Cell Analyzer 6000 (GE). Viability was also measured on day 10 by using the CellTiter-Glo 3D Cell Viability Assay (Promega) in accordance with the manufacturer’s protocol, with the results being recorded in luminescence units.

### Reactome, gene ontology (GO) analysis, and Kaplan-meier survival plots

All genes that correlated to depleted sgRNAs from the negative-selection (drop-out) CRISPR screen were filtered at a maximum *P*-value of 0.05. The scores of the remaining 1073 genes were transformed such that the highest value was represented as 1 in order to assign weight. All statistically significant genes were verified to have a weight greater than 0, and the list and corresponding weights were loaded into Enrichr [18] for downstream analysis. Enrichment analysis of pathways were obtained by selecting the Reactome Pathways 2016 and EMBL GO Biological Process databases. For transcription factor enrichment analysis, the eXpression2Kinases tool [19] was used with all default settings. The cBioPortal tool [20, 21] was used to measure expression of *PSMA6* across available patient samples from the TCGA Research Network: http://cancergenome.nih.gov/. Kaplan-Meier survival plot was generated using Kaplan-Meier plotter using Pan-cancer RNAseq dataset [22].

### Statistical analysis

MAGeCK-VISPR [16] was used to rank and sort gRNAs by *P*-value and/or FDR (Additional file 3). Data from at least three independent replicated experiments were quantitatively analyzed by two-way ANOVA with the Sidak multiple comparisons test or by Student’s 2-tailed *t*-test, using GraphPad Prism 7.0 software, as indicated. All data are represented as the mean ± SD.

## Results

### Genome-wide CRISPR screen and hit validation

**Fig. 1:**
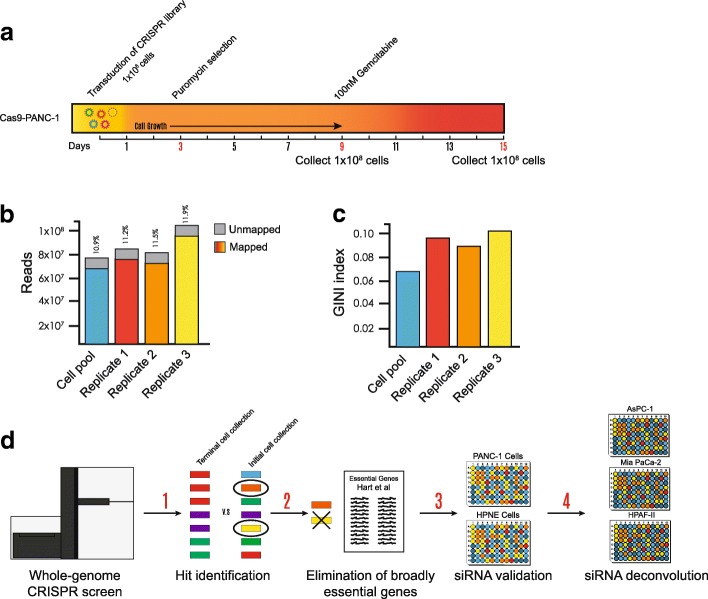
Genome-wide CRISPR screen overview. **a** A negative-selection screen was conducted in CAS9–PANC-1 cells. The cells were treated with 100 nM (IC_10_) of gemcitabine from day 9 post transduction and collected for genomic DNA analysis on day 15. **b** Total reads, both mapped and unmapped. **c** Gini index (a measurement of inequality, with 1 being the most unequal) of the starting cell population and the three replicates of the dropout screen. **d** Candidate genes were identified after a dropout CRISPR screen (1) and the elimination of previously identified pan-essential genes (2). Resultant hits were further validated with pooled siRNA screens in PANC-1 and HPNE cells (3), and four individual siRNAs were deconvoluted and further validated in AsPC-1, Mia PaCa-2, and HPAF-II cells (4)

We conducted a negative-selection (drop-out) genome-wide CRISPR screen to uncover novel essential genes and/or genes that might sensitize pancreatic cancer cells to the current frontline chemotherapeutic agent, gemcitabine. We transduced the Brunello CRISPR library (on day 0) and allowed cell outgrowth for a further 9 days under puromycin selection. The stable cells were then treated with 100 nM (the approximate IC_10_) [23–25] of gemcitabine (on day 9) for an additional 6 days (to day 15) (Fig. 1a; Additional file 1a). Next-generation sequencing (NGS) was performed on 100-bp amplicons with upwards of 8 × 10^7^ reads, of which approximately 90% were mapped to the gRNA library (Fig. 1b). Our sequencing also revealed an obvious change in the representation of gRNAs as measured by the GINI index [26], which is a measurement of inequality (Fig. 1c), and demonstrated a selection event. After performing NGS, we identified drop-out hits by using MAGeCK-VISPR software; a complete list of hits can be found in Additional file 3. The hits were prioritized by eliminating previously identified essential genes that had been shown to be critical in most cell lines by Hart et al. [10]. We hypothesized that those previously identified essential genes would not be of interest because of their lack of specificity for PDAC cell lines. The resulting top 100 (approximately) hits were then screened with four pooled siRNAs per gene in PANC-1 and hTert HPNE cells. HPNE cells are an intermediary cell line formed during acinar-to-ductal metaplasia that we used as our noncancerous pancreas cell line control. The siRNAs that showed preferential essentiality were deconvoluted and further tested in the AsPC-1, Mia PaCa-2, and HPAF-II cell lines (Fig. 1d).

Interestingly, a Gene Ontology (GO) analysis and a pathway analysis from the Reactome database revealed cell-cycle genes to be significantly enriched among the top depleted sgRNAs in our screen (Additional file 4a–d). Additionally, a transcription factor enrichment analysis revealed that MYC is an upstream transcription factor for the genes identified in the negative-selection screen (Additional file 4e).

### Screening for pancreatic cancer specific hits

**Fig. 2:**
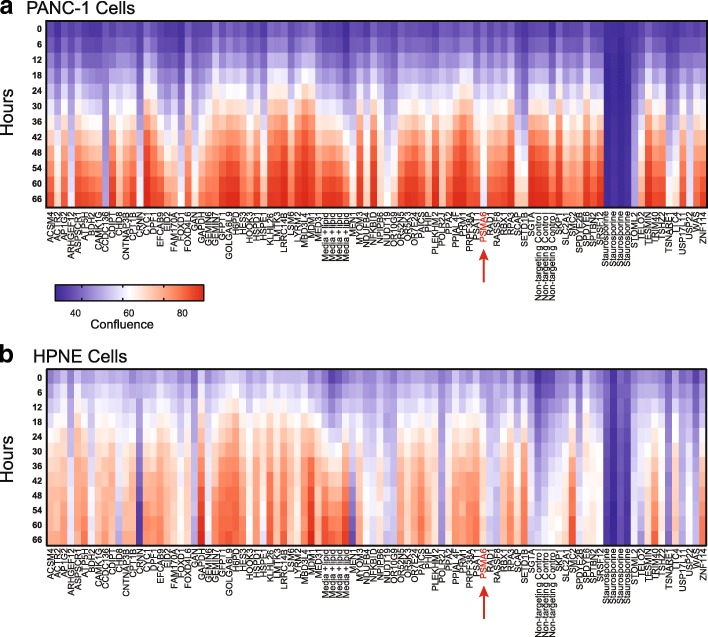
Heat map and hit validation using a pool of siRNAs in PANC-1 and HPNE cells. Heat map showing cell confluence (measured with the IncuCyte Live Cell Analysis System) after transfection of siRNA into **a** PANC-1 cells and **b** HPNE cells. *PSMA6* is shown in red text and indicated by an arrow. The cell confluence is color coded: blue represents low confluence and red represents high confluence, as measured by live-cell imaging over 66 h. Samples were normalized to controls treated with 1 μM staurosporine (as a positive control for cell death) and lipid reagent alone

We conducted a siRNA screen with a pool of four individual siRNAs in an effort to validate the top 100 (approximately) hits from the genome wide CRISPR screen and to identify hits that selectively affected PANC-1 cells as compared to HPNE cells, which are a model of noncancerous pancreatic tissue. In both pooled siRNA screens, we monitored the cells for a short period (66 h post transfection) to look for the most potent essential genes. Longer, 5-day siRNA screens for these same gene hits revealed that almost all the siRNAs targeting these genes elicit some degree of growth defect (Additional file 5a). The pooled siRNA screen in PANC-1 cells revealed several siRNAs, including those targeting *ARHGEF12*, *CCDC136*, *CRNN*, *FOXD1*, *NUDT19*, *PSMA6*, *STOML2*, *TSNARE1*, and *USP22*, that significantly impede growth (Fig. 2a). Similarly, the pooled siRNA screen in HPNE cells revealed several potent siRNAs, some of which are unique to HPNE, including those targeting *MEN1* and *CRNN* (Fig. 2b). Interestingly, HPNE cells appear to be sensitive to the non-targeting siRNA alone, most likely because one or more of the individual non-targeting siRNAs within the pool has off-target toxic effects within these cells. However, we were most interested in the targets that have little or no phenotypic effect in HPNE cells but are highly potent in PANC-1 cells. Given this criterion, we identified *CCDC136* and *PSMA6* as potential selective targets (Fig. 2b).

### siRNA deconvolution and cell line specificity

**Fig. 3:**
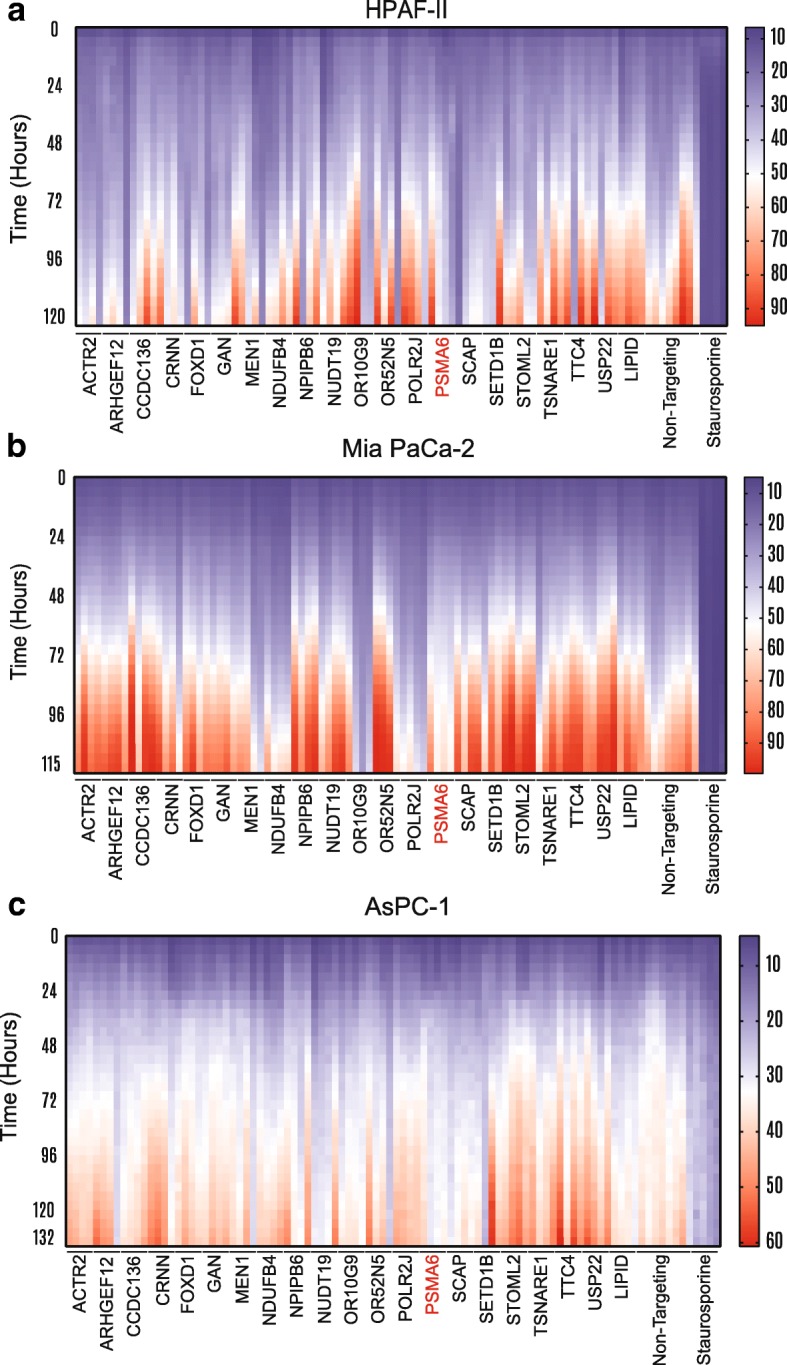
Heat map and deconvolution of four individual siRNAs. Heat map showing cell confluence after transfection of four individual siRNAs per gene in **a** HPAF-II, **b** Mia PaCa-2, and **c** AsPC-1 cells. *PSMA6* is shown in red. The cell confluence is color coded: blue indicates low confluence and red indicates high confluence, as measured by live-cell imaging over the specified number of hours. Samples were normalized to controls treated with 1 μM staurosporine (as a positive control for cell death) and lipid reagent alone

To confirm our observations with siRNA-mediated knockdown, we deconvoluted all pooled siRNAs that showed any potent effect in PANC-1 cells by testing the four individual siRNAs independently. The pool of four individual siRNAs was deconvoluted to validate the phenotype and to limit the probability of the phenotype’s being caused by an off-target effect. For additional validation, we tested whether our observations were cell-line specific. To this end, we measured the resulting growth characteristics with live-cell imaging and quantification of four individual siRNAs per target in three different PDAC cell lines: HPAF-II (Fig. 3a), Mia PaCa-2 (Fig. 3b), and AsPC-1 (Fig. 3c). We clearly showed that there are cell line specific on-target effects; for example, the siRNA targeting *CCDC136* is very potent in PANC-1 cells but has limited or no effect in other tested pancreatic cancer cell lines, which includes HPAF-II, Mia PaCa-2, and AsPC-1 cells. Thus, *CCDC136* knockdown may exploit a unique molecular mechanism in PANC-1 cells that is not present in all models. It is also noteworthy that the effects of siRNA knockdown of *SCAP*, *ARHGEF12*, and *OR10G9* on cell growth vary among these cell lines, whereas the siRNA targeting *MEN1* was toxic in all the tested cell lines, including HPNE (Figs. 2a and b, 3a–c). The siRNA targeting *PSMA6* (siPSMA6) showed similar toxicity siRNA targeting *MEN1*, but noncancerous HPNE cells were moderately resistant to it (Figs. 2b, 3a–c, Additional file 5b). Movies illustrating the phenotype observed in HPNE and PANC-1 cells can be found in Additional file 6.

### *PSMA6* inhibition results in apoptosis and reduced spheroid formation

**Fig. 4:**
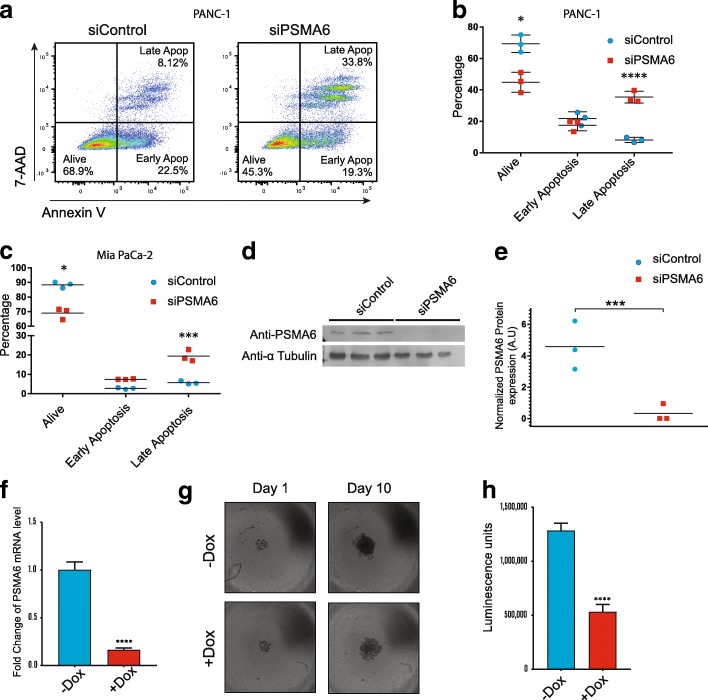
*PSMA6* knockdown results in apoptosis and reduced spheroid formation. **a** Flow cytometric analysis and quantification of 7-AAD and annexin V staining in **b** PANC-1 cells and **c** Mia PaCa-2 cells 72 h post transfection with siControl (non-targeting siRNA) or siPSMA6. **d** Western blot of siControl and siPSMA6 samples probed with PSMA6 and tubulin antibodies after 72 h of siRNA treatment. **e** Quantification of the western blot with the samples normalized to tubulin expression levels. **f** PSMA6 levels after 500 nM doxycycline treatment in stable PANC-1 cells expressing a tet-on *PSMA6* shRNA sequence (tet-on shPSMA6). **g** Spheroid formation at days 1 and 10 after doxycycline induction in tet-on shPSMA6 PANC-1 cells and **h** quantification at day 10 with a cell viability assay (CellTiter-Glo). (*P = 0.05; ***P = 0.01, ****P = 0.0001)

We hypothesized that *PSMA6* inhibition resulted in cellular apoptosis because the PSMA6 protein was a critical member of the proteasome. To test this, we ran flow cytometry on PANC-1 cells after 72 h of treatment with 25 nM siPSMA6 (Fig. 4a). We identified a clear shift in late apoptosis, with a subpopulation of siPSMA6-treated PANC-1 and Mia PaCa-2 cells exhibiting a significant upward shift in late apoptotic events when compared to cells treated with siControl (non-targeting siRNA) (Fig. 4b and c; Additional file 7). We confirmed PSMA6 protein knockdown with a western blot (Fig. 4d and e).

To perform long-term spheroid assays, we made PANC-1 cells that stably expressed shRNA against *PSMA6* (shPSMA6), using the tet-on system inducible by doxycycline (Dox) to circumvent the limitation of transient siRNA. We validated the shPSMA6-stable cells and were able to achieve knockdown of *PSMA6* with greater than 80% efficiency (Fig. 4f). Using the tet-on shPSMA6 cells, we conducted 10-day spheroid assays in ultra-low–attachment round-bottom plates and monitored the spheroids with imaging followed by a terminal viability assay (CellTiter-Glo). We found that the cells with silenced PSMA6 were significantly smaller and visually less dense (Fig. 4g); furthermore, total cell viability, as measured by the CellTiter-Glo luminescence, was decreased by approximately 60% in cells where PSMA6 expression was blocked (Fig. 4h). As expected, the Cancer Genome Atlas shows *PSMA6* is expressed in human PDAC samples and has a relatively low mutation rate (Additional file 8a). And in support of our spheroid assays, we generated a Kaplan-Meier survival curve using KM plotter [22] which shows PDAC patients with high expression of *PSMA6* having a significantly shorter overall survival rate (*P =* 0.0009) (Additional file 8b)**.**

### Bortezomib is extremely potent in PANC-1 cells and results in rapid apoptosis

**Fig. 5:**
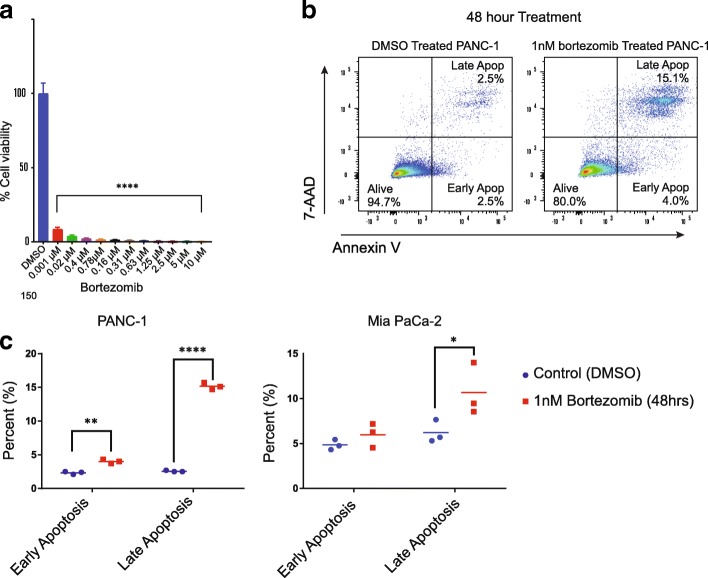
Bortezomib inhibition of the proteasome in PANC-1 cells results in decreased viability and cell death. **a** Bortezomib dosage in PANC-1 cells and percentage viability after 96 h of treatment. **b** Representative flow cytometry panels of PANC-1 cells and **c** quantification of PANC-1 cells and Mia PaCa-2 cells treated with 1 nM bortezomib for 48 h then stained with 7-AAD and Annexin V to assess cell apoptosis. (*P = 0.05, **P = 0.01; ****P = 0.0001)

After verifying the necessity of PSMA6 for PANC-1 and Mia PaCa-2 cell survival, we next set out to interrogate the susceptibility of PANC-1 and Mia PaCa-2 cells to disruption of the broader biological pathways that involve PSMA6 expression. Because PSMA6 is a critical member of the proteasome, we hypothesized that PANC-1 and Mia PaCa-2 cells would be sensitive to therapeutic inhibition of the proteasome. To that end, we treated both cell lines with bortezomib, a current FDA-approved proteasome inhibitor that binds the catalytic site of the 26S proteasome and has also been shown to interact with the β subunits of the 20S proteasome [27]. Bortezomib was chosen in an effort to phenocopy PSMA6 knockdown, with the caveat that PSMA6 is just a member of the much larger proteasome complex that is inhibited by bortezomib. After treatment with bortezomib we found that it significantly increased cellular death. In fact, sub–1 nM concentrations decreased PANC-1 cell viability by approximately 90% after a 5-day treatment (Fig. 5a). We also treated PANC-1 and Mia PaCa-2 cells with 1 nM bortezomib for 48 h, then stained the cells with 7-AAD and annexin V and performed flow cytometry, revealing that bortezomib treatment significantly and rapidly induced late apoptosis (Fig. 5b and c; Additional file 9).

## Discussion

PDAC is an aggressive cancer that has a poor prognosis because of various factors, including the poor treatment options available [28]. Although various chemotherapeutic treatment combinations are being tested [29], most advances in PDAC treatment have been in the areas of early detection and surgical resection of tumors, and there has been little progress in developing effective treatment options for advanced cases [30]. Therefore, it is vitally important to develop novel treatments for this cancer.

In the present study, we aimed to uncover genes required for PDAC cell growth to potentially reveal novel targets for developing effective anticancer agents. To achieve this, we conducted a genome-wide CRISPR screen in PANC-1 cells, together with validation siRNA screens in several PDAC cell lines. We were unable to achieve both of our original goals- to identify essential genes and genes that would sensitize PDAC cells to gemcitabine. One potential hypothesis is the redundancy of gemcitabine transporters in PDAC cells [13]. With that said, we were still able to identify a variety of candidate essential genes in several PDAC cell lines.

Gene Ontology (GO) analysis and a pathway analysis revealed that cell-cycle genes were disproportionally enriched among the gene hits and that MYC is a probable upstream transcription factor for many of the identified gene hits. MYC deregulation and activation are involved in many PDAC models, and MYC has been hypothesized to be a potential novel therapeutic [31]. Additionally, MYC has been implicated in chemosensitization to cisplatin and paclitaxel (Taxol), probably through cell-cycle regulation [32, 33].

We focused on *PSMA6* because it was the top hit that was not a cycle-regulator or a target of MYC and is an important component of a potentially targetable pathway. *PSMA6,* which encodes a subunit of the proteasome, is ubiquitously expressed; although normal pancreas has low (compared to other normal tissues) mRNA expression [34]. And *PSMA6* is expressed within human PDAC samples and is largely unaltered without mutations (Additional file 8a). Furthermore, we identified *PSMA6* as an essential gene in all the tested PDAC cell lines: PANC-1, Mia PaCa-2, AsPC-1, and HPAF-II. Interestingly, when tested in the noncancerous HPNE cells, siPSMA6 appeared to have little effect, and cells treated with this siRNA grew similarly to controls treated with a non-targeting siRNA (Fig. 2b; Additional file 5b; Additional file 6). All this data taken together indicates that the knockdown of PSMA6 and subsequent very low levels of PSMA6 expression results in cellular death in PDAC cell models. Additionally and in support of PSMA6’s affect in PDAC cells, *PSMA6* also has a similar phenotype in lung cancer and is also dispensable in normal lung tissue [35].

One major caveat is the fact that all this work is done in vitro. In an attempt to model in *an* in vivo environment we decided to use a spheroid assay [36]. By using spheroid assays and shRNA against *PSMA6*, we further validated this gene as being essential for PDAC growth in a 3D environment, further indicating that *PSMA6* may be a viable therapeutic target that warrants further in vivo study. Of significant note, a Kaplan-Meier survival of curve shows high expression of *PSMA6* is associated with a shorter overall survival in PDAC patients (*P =* 0.0009) (Additional file 8b).

*PSMA6* has also been shown to have an oncogenic role in several cancer types [35, 37, 38]. And more broadly, the ubiquitin-proteasome degradation pathway has been shown to be critical for cell survival and proliferation. Many cancers have been shown to have an increased sensitivity to perturbations within the proteasome pathway through a variety of mechanisms including dysregulation of short-lived cell cycle proteins and the accumulation of misfolded proteins [39, 40]. Bortezomib was developed to inhibit the proteasome and is approved for treating multiple myeloma and mantle cell non-Hodgkin lymphoma [41]. Bortezomib induces apoptosis in pancreatic cancer cells, probably through a host of pathways, including ceramide formation and ER stress [42–44]. Consistent with these studies and with our data on inhibition of *PSMA6*, we have shown that PDAC cells are sensitive to bortezomib treatment. Furthermore, bortezomib treatment results in the rapid onset of apoptosis, with a large population of cells entering late apoptosis within 48 h. It is important to note that bortezomib also binds to the β subunits of the 20S proteasome. Variants of the β subunits, specifically β5 [27], have been associated with resistance in vitro [45], however these variants are not seen in vivo [46]. Thus the effects of bortezomib may not be due solely to the inhibition of PSMA6. Additionally, the effects of bortezomib on pancreatic cancer have been shown to be limited to in vitro assays and observable only in combination treatment with other agents, such as gemcitabine [47–49] (Clinical Trial # NCT00052689).

## Conclusion

We have identified several potential new essential genes for PDAC through a screening pipeline. This pipeline included a genome-wide CRISPR screen followed by multiple siRNA screens in several PDAC cell models (PANC-1, Mia PaCa-2, HPAF-II, and AsPC-1) and in a noncancerous cell model (HPNE). Lastly, we validated our top identified hit, *PSMA6*, by using siRNA and inducible shRNA to show that inhibition of this gene induces apoptosis and results in significantly reduced cell viability. Our in vitro work and the Kaplan-Meier plot (shows a negative correlation between *PSMA6* mRNA expression and overall survival) both provide compelling evidence that *PSMA6* plays a significant oncogenic role. Future work needs to be done to fully assess *PSMA6*s in vivo oncogenic role. Lastly, we propose future work into the development of a specific PSMA6 inhibitor that could be used in combination with bortezomib or other chemotherapeutic drugs to treat PDAC. We will also pursue the other gene hits identified in our screening pipeline.

## Additional files

Additional file 1: a) Detailed overview of the CRISPR screen methodology, illustrating the timeline and replicates of samples. b) Western blot analysis of CAS9 expression in PANC-1 and Mia PaCa-2 cells. (PDF 1199 kb)

Additional file 2: Sequencing primers and barcodes for PCR amplification and Next-Gen Sequencing of amplicons. (XLSX 12 kb)

Additional file 3: Ranked gene list results from the negative selection CRISPR screen. (XLSX 1554 kb)

Additional file 4: a) Gene Ontology (GO) analysis and b) corresponding bar chart highlighting significantly enriched terms. c) Pathway analysis from Reactome (2018) and d) corresponding bar charts highlighting the significantly enriched pathways. e) Transcription Factor Enrichment Analysis of the top gene hits identified from the negative (dropout) CRISPR screen. (PDF 2361 kb)

Additional file 5: a) siRNA secondary screen measuring cell viability with CellTiter-Glo. Data were normalized to controls (*PLK1* set at 100% growth inhibition and lipid transfection reagent set to 0%) are presented as the percentage growth inhibition. *PSMA6* is shown in red on the x-axis. b) Quantification of non-targeting siRNA (siNT), siPSMA6, and staurosporine (staur.) treated AsPC-1, HPAF-II, Mia PaCa, and HPNE cells at end-point confluence as shown in the heat maps found in Figs. 2b and 3a**-**c. (**P = 0.02; ***P = 0.01, ****P = 0.0001, ns = not significant) (PDF 1394 kb)

Additional file 6: (Movies). Non-targeting siRNA (movie 1) and siPSMA6 (movie 2) were transfected at 25 nM into PANC-1 cells and images were acquired every 4 h with an IncuCyte Live Cell Analysis System. Non-targeting siRNA (movie 3) and siPSMA6 (movie 4) were transfected at 25 nM into HPNE cells and images were acquired every 4 h with an IncuCyte Live Cell Analysis System. (ZIP 34546 kb)

Additional file 7: Complete flow cytometry panel for 7-AAD and Annexin V staining in Mia PaCa-2 and PANC-1 cells 72 h post transfection with siControl (non-targeting siRNA) or siPSMA6 (see Fig. 4b and c). (PDF 704 kb)

Additional file 8: a) *PSMA6* expression query with cBioPortal tool from the TCGA Research Network. b) Kaplan-Meier plot of high and low *PSMA6* expression in PDAC patient samples and overall survival. (PDF 29422 kb)

Additional file 9: Complete flow cytometry panel for 7-AAD and Annexin V staining in Mia PaCa-2 and PANC-1 cells after 48 h of treatment with 0.001 μM bortezomib or DMSO control (controls 1–3) (see Fig. 5b–d). (PDF 743 kb)

## Abbreviations

ARHGEF12: Rho Guanine Nucleotide Exchange Factor 12
CCDC136: Coiled-Coil Domain Containing 136
CRNN: Cornulin
Dox: Doxycycline
ER: Endoplasmic reticulum
FOXD1: Forkhead Box D1
LD50: Lethal dose 50
MEN1: Menin 1
NUDT19: Nudix Hydrolase 19
OR10G9: Olfactory Receptor Family 10 Subfamily G Member 9
PDAC: Pancreatic ductal adenocarcinoma
PSMA6: Proteasome Subunit Alpha 6
SCAP: SREBF Chaperone
STOML2: Stomatin Like 2
TSNARE1: T-SNARE Domain Containing 1
USP22: Ubiquitin Specific Peptidase 22
