# Supplementary figures and images for: Genome-wide CRISPR screen reveals PSMA6 to be an essential gene in pancreatic cancer cells
Source: BMC Cancer. 2019 Mar 21;19:253. doi: 10.1186/s12885-019-5455-1 (PMC6429770; doi:10.1186/s12885-019-5455-1)

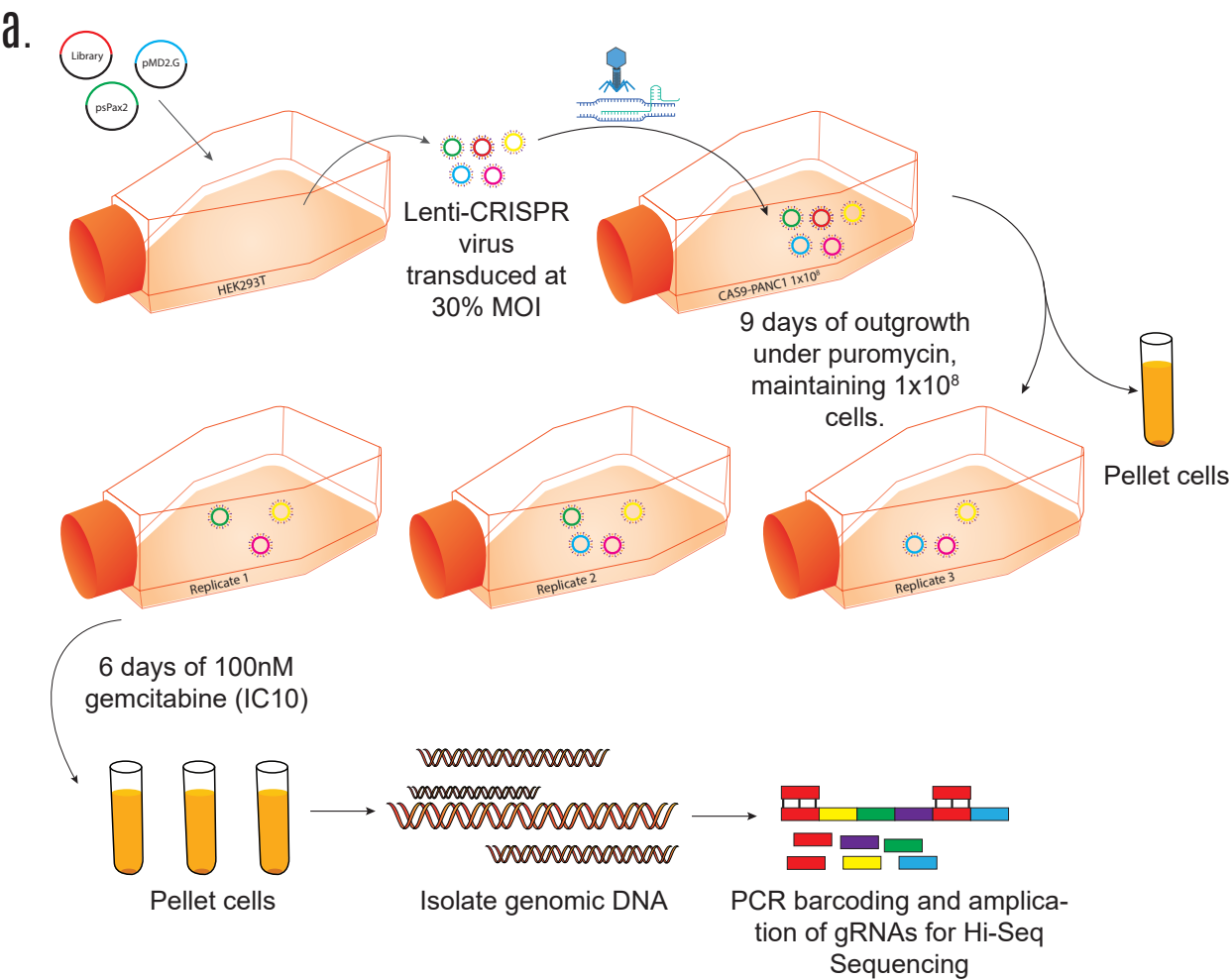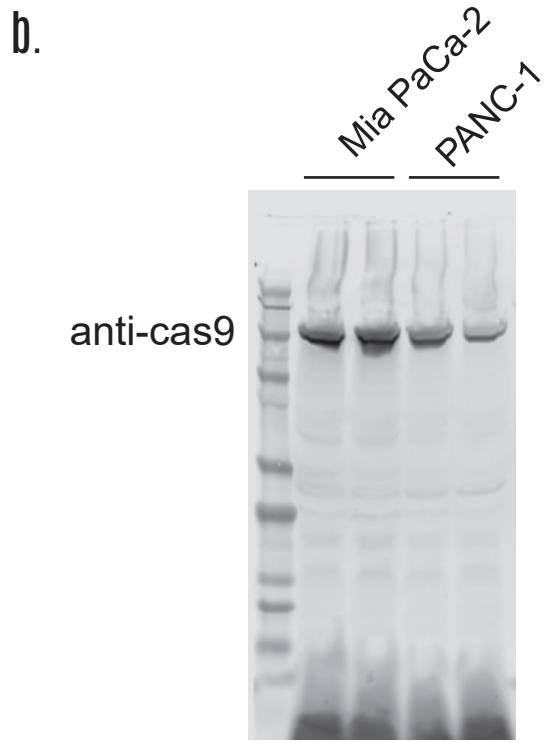

Supplement: Supplementary file 1 — a) Detailed overview of the CRISPR screen methodology, illustrating the timeline and replicates of samples. b) Western blot analysis of CAS9 expression in PANC-1 and Mia PaCa-2 cells. (PDF 1199 kb) [file 12885_2019_5455_MOESM1_ESM.pdf]

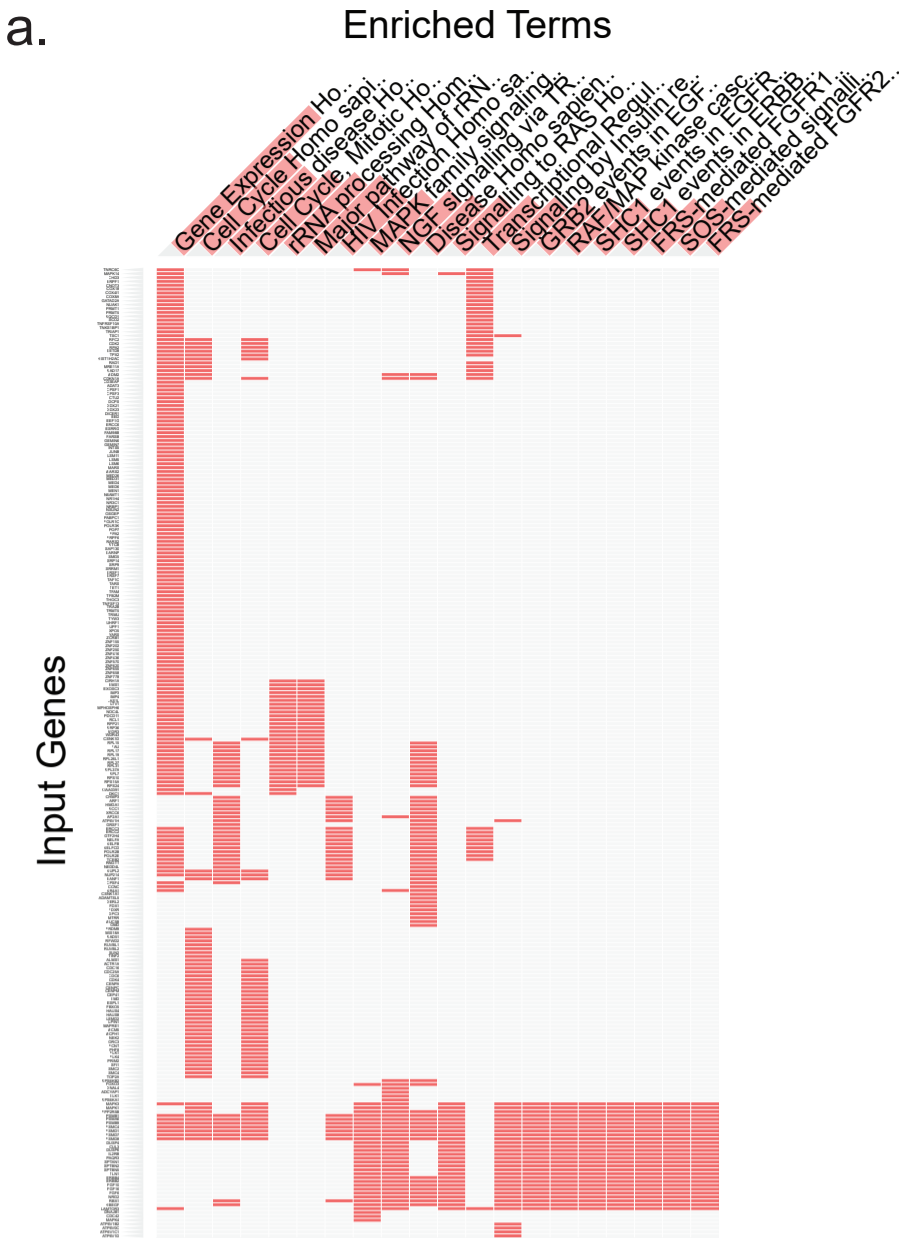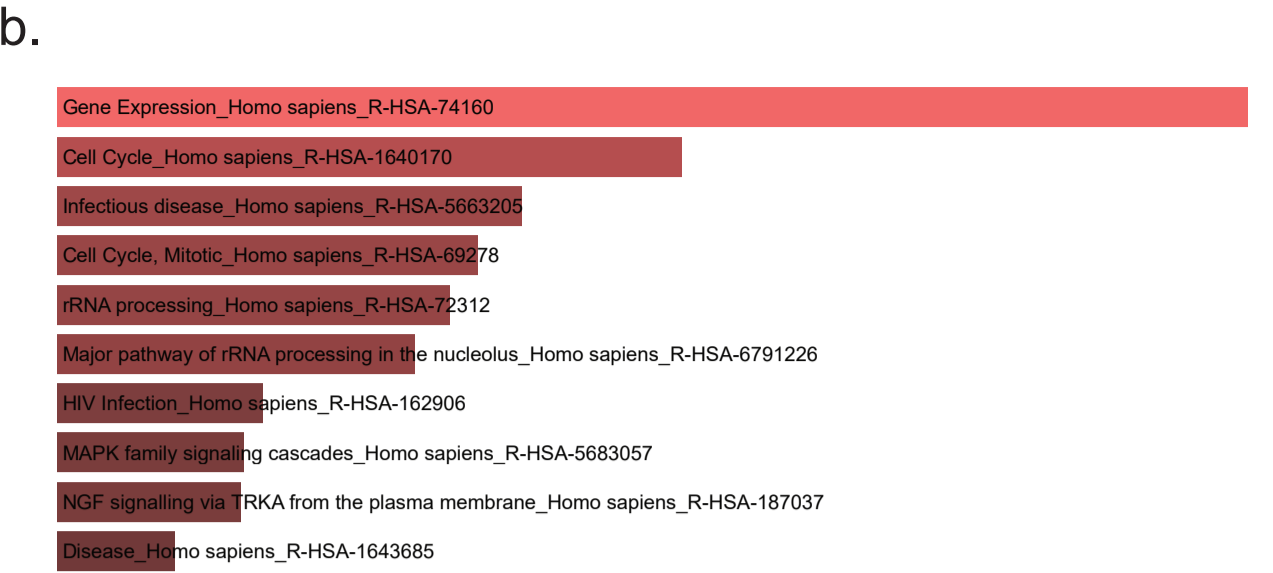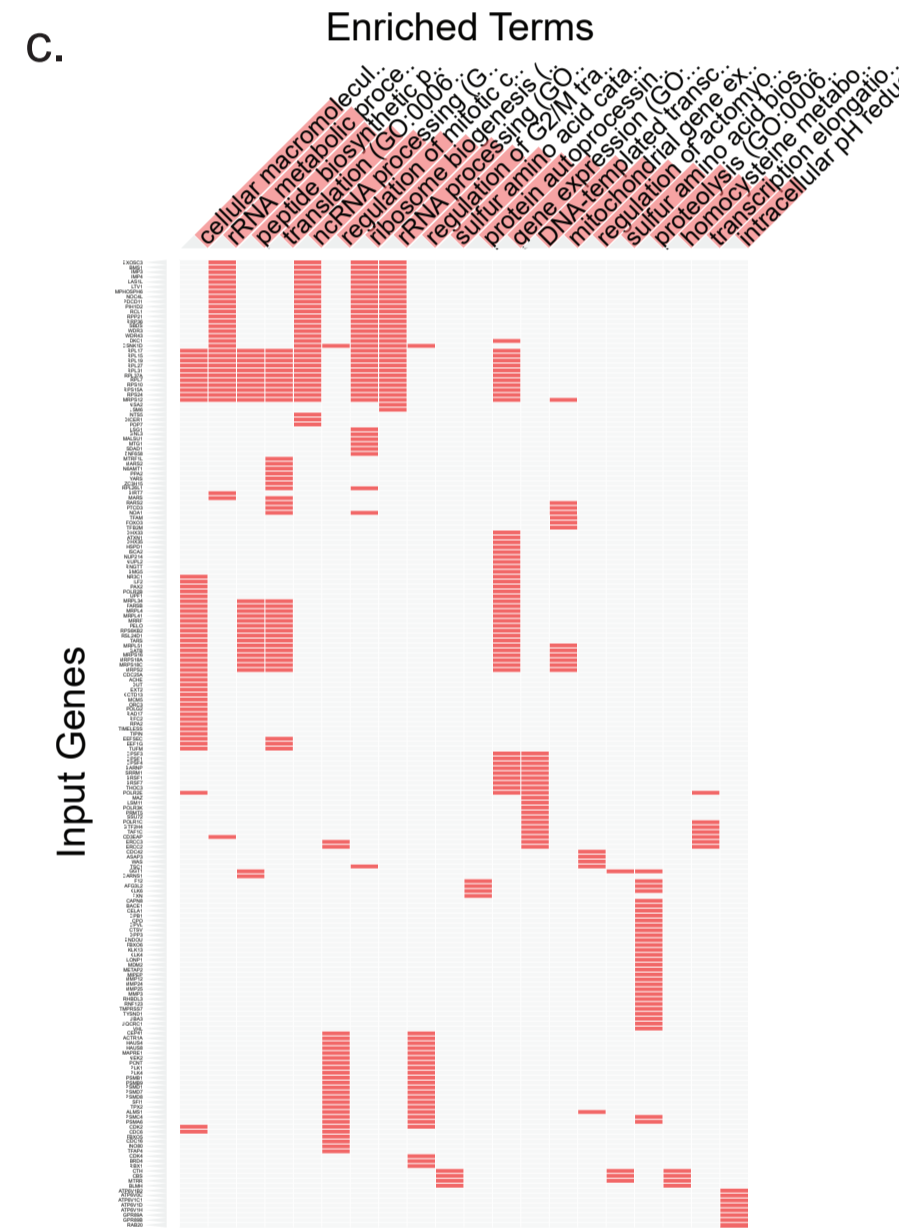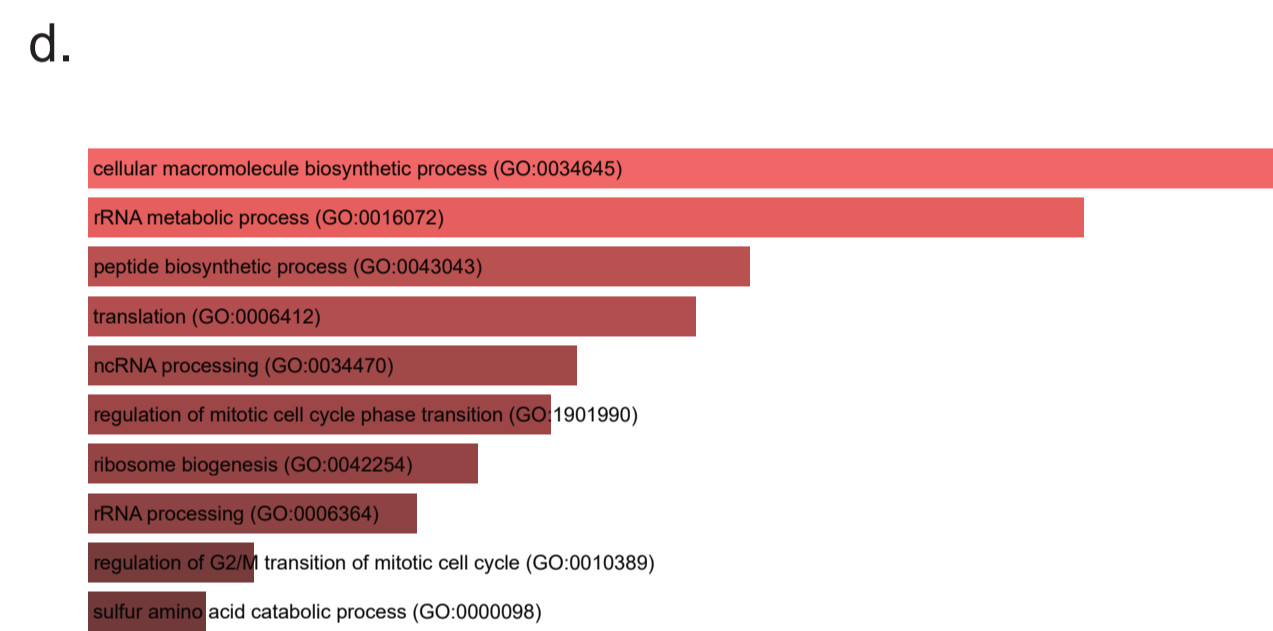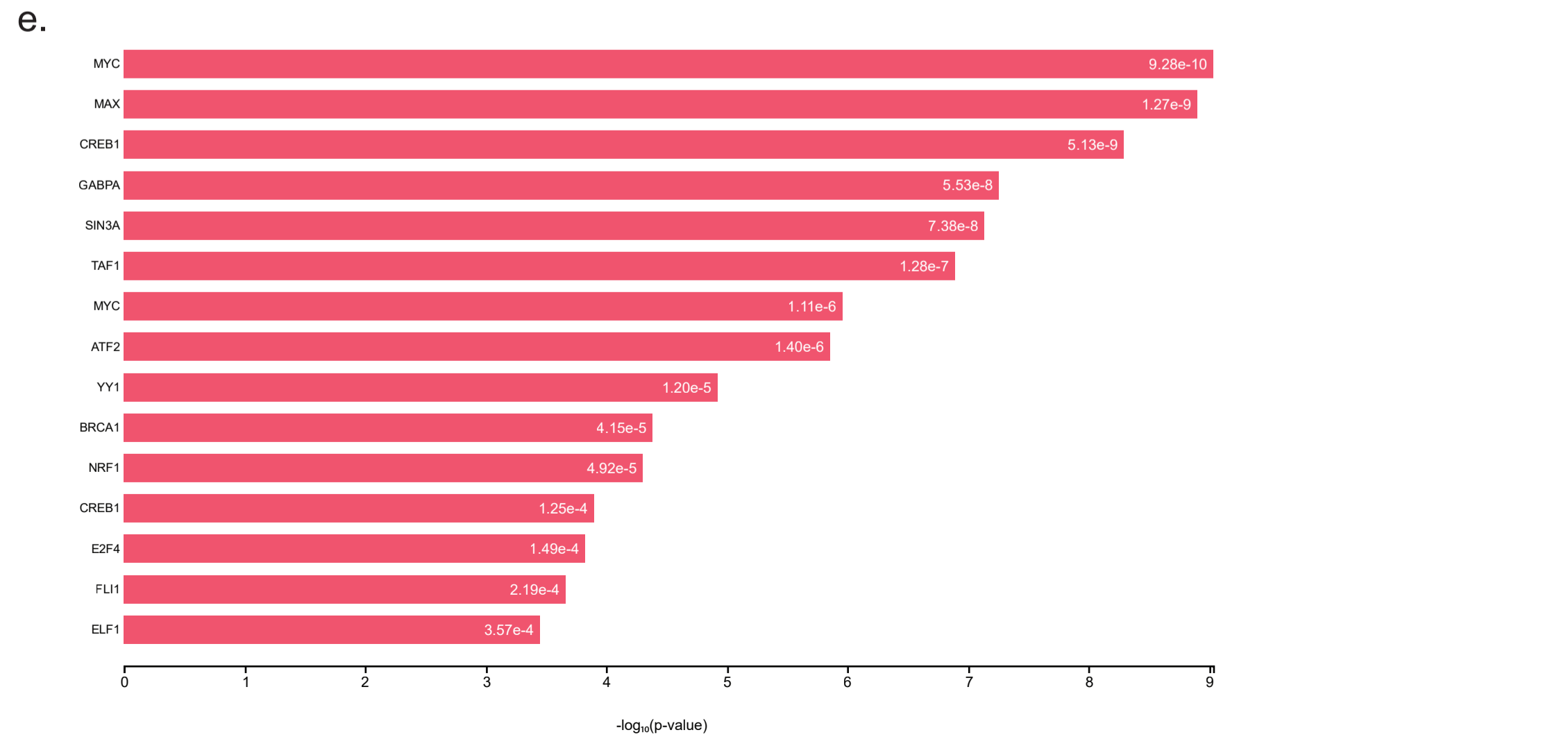

Supplement: Supplementary file 4 — a) Gene Ontology (GO) analysis and b) corresponding bar chart highlighting significantly enriched terms. c) Pathway analysis from Reactome (2018) and d) corresponding bar charts highlighting the significantly enriched pathways. e) Transcription Factor Enrichment Analysis of the top gene hits identified from the negative (dropout) CRISPR screen. (PDF 2361 kb) [file 12885_2019_5455_MOESM4_ESM.pdf]

a.

PANC-1 Cells

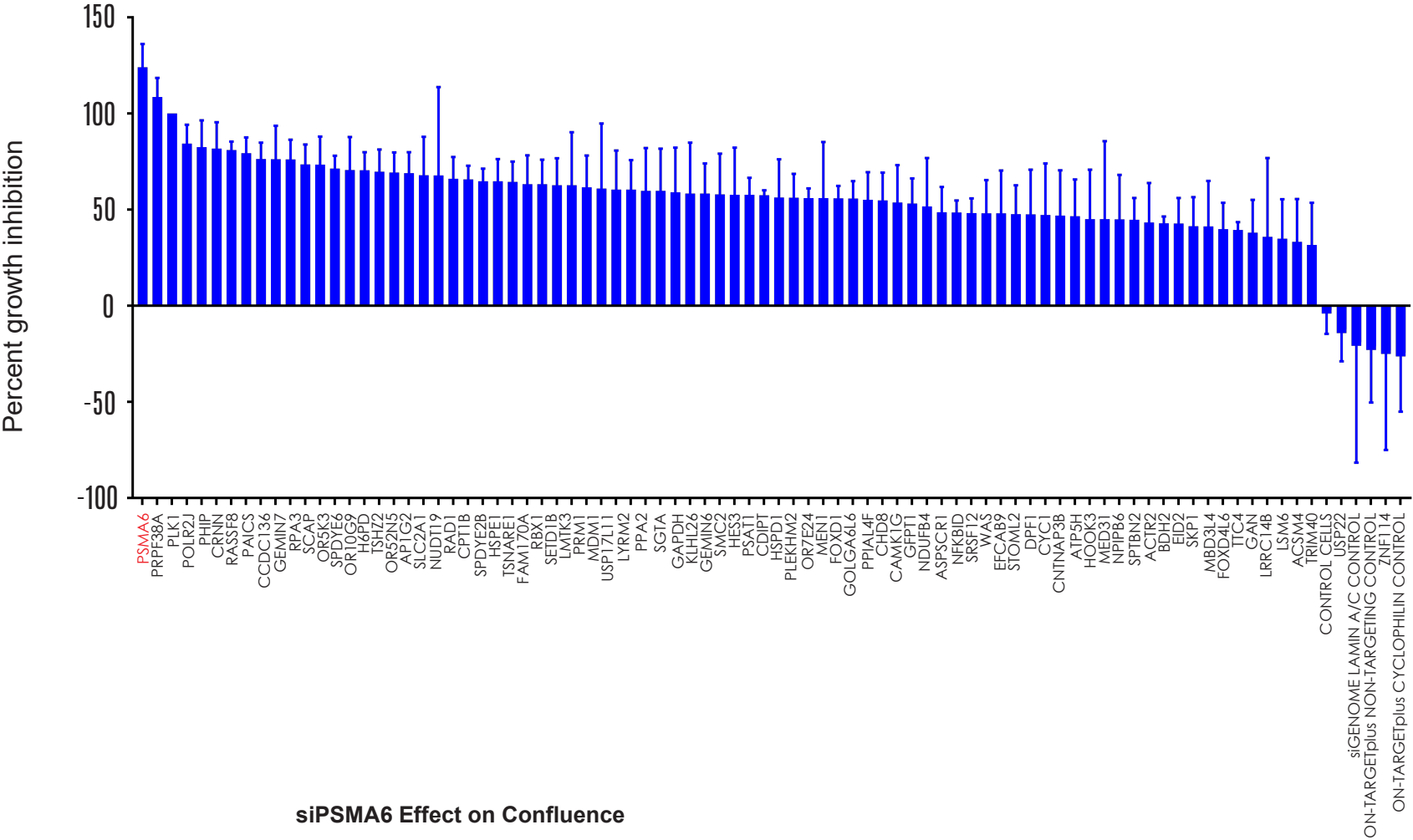

b.

siPSMA6 Effect on Confluence

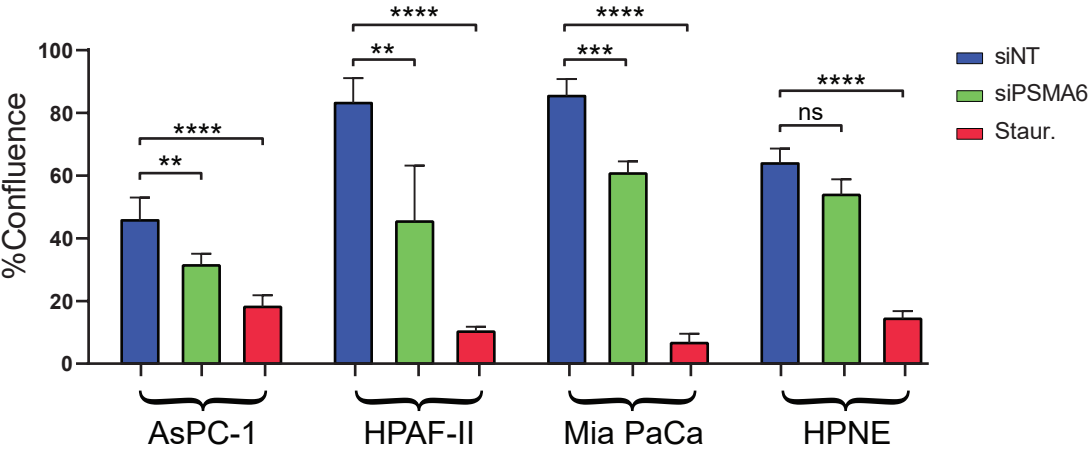

Supplement: Supplementary file 5 — a) siRNA secondary screen measuring cell viability with CellTiter-Glo. Data were normalized to controls (PLK1 set at 100% growth inhibition and lipid transfection reagent set to 0%) are presented as the percentage growth inhibition. PSMA6 is shown in red on the x-axis. b) Quantification of non-targeting siRNA (siNT), siPSMA6, and staurosporine (staur.) treated AsPC-1, HPAF-II, Mia PaCa, and HPNE cells at end-point confluence as shown in the heat maps found in Figs. 2b and 3a-c. (**P = 0.02; ***P = 0.01, ****P = 0.0001, ns = not significant) (PDF 1394 kb) [file 12885_2019_5455_MOESM5_ESM.pdf]

7-AAD vs Annexin V

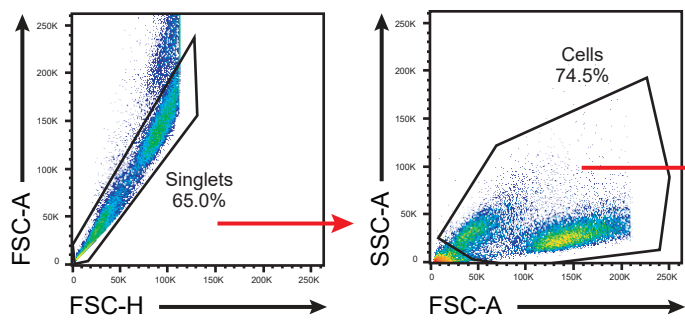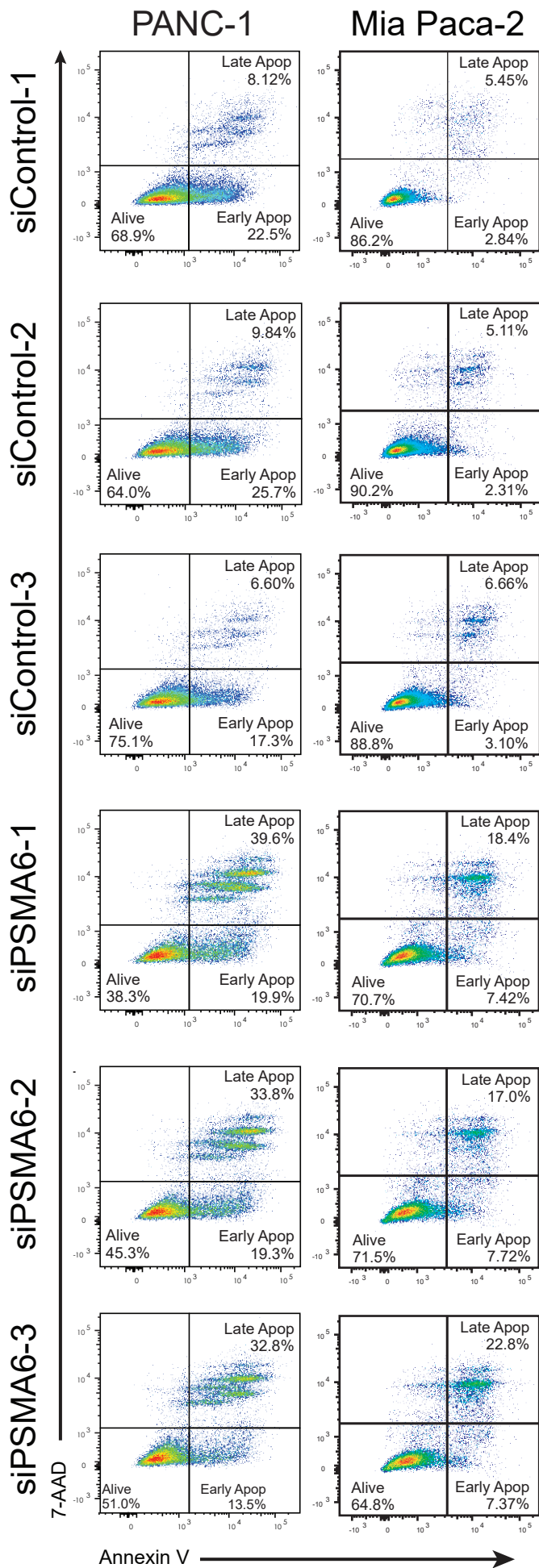

Supplement: Supplementary file 7 — Complete flow cytometry panel for 7-AAD and Annexin V staining in Mia PaCa-2 and PANC-1 cells 72 h post transfection with siControl (non-targeting siRNA) or siPSMA6 (see Fig. 4b and c). (PDF 704 kb) [file 12885_2019_5455_MOESM7_ESM.pdf]

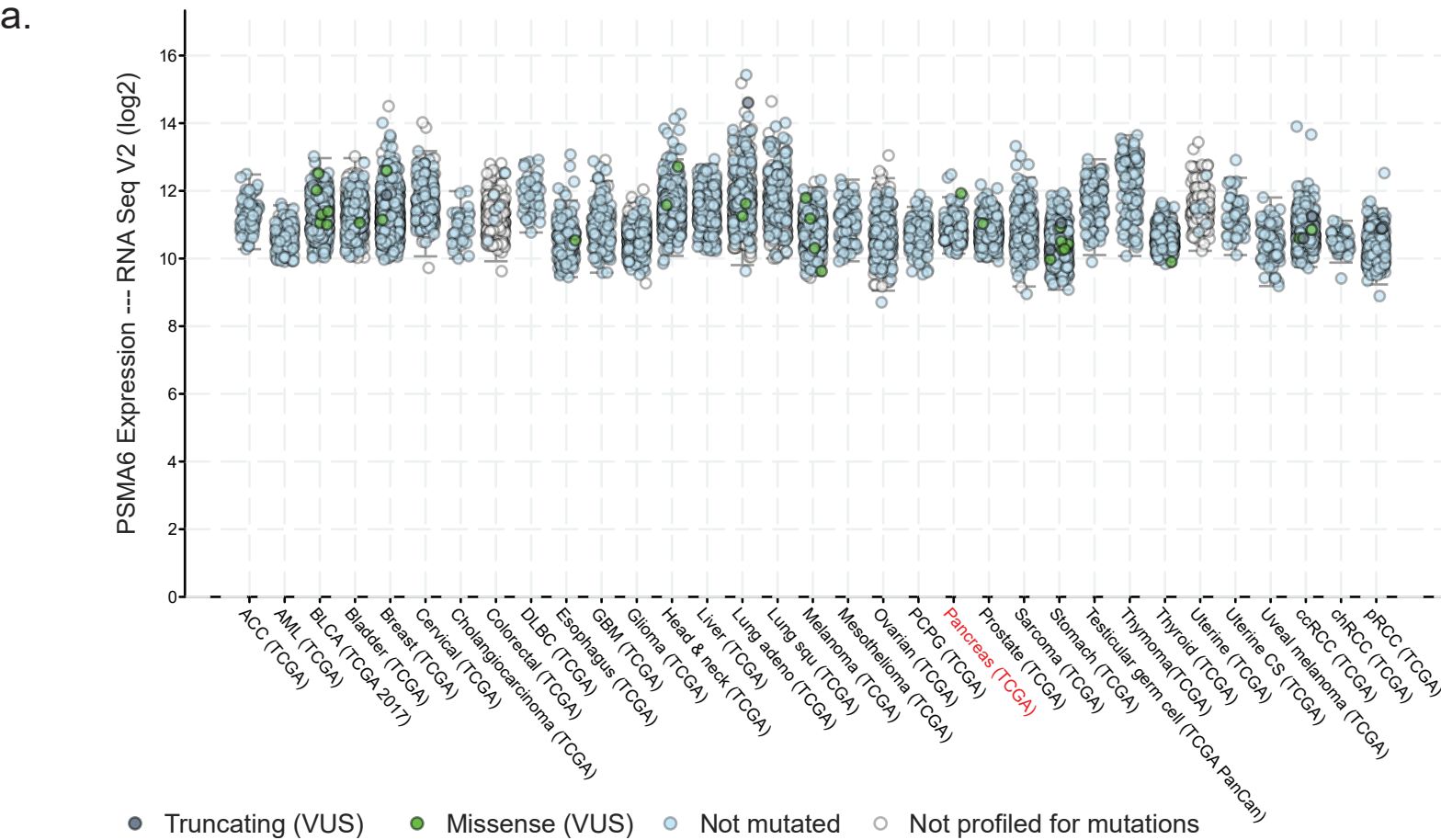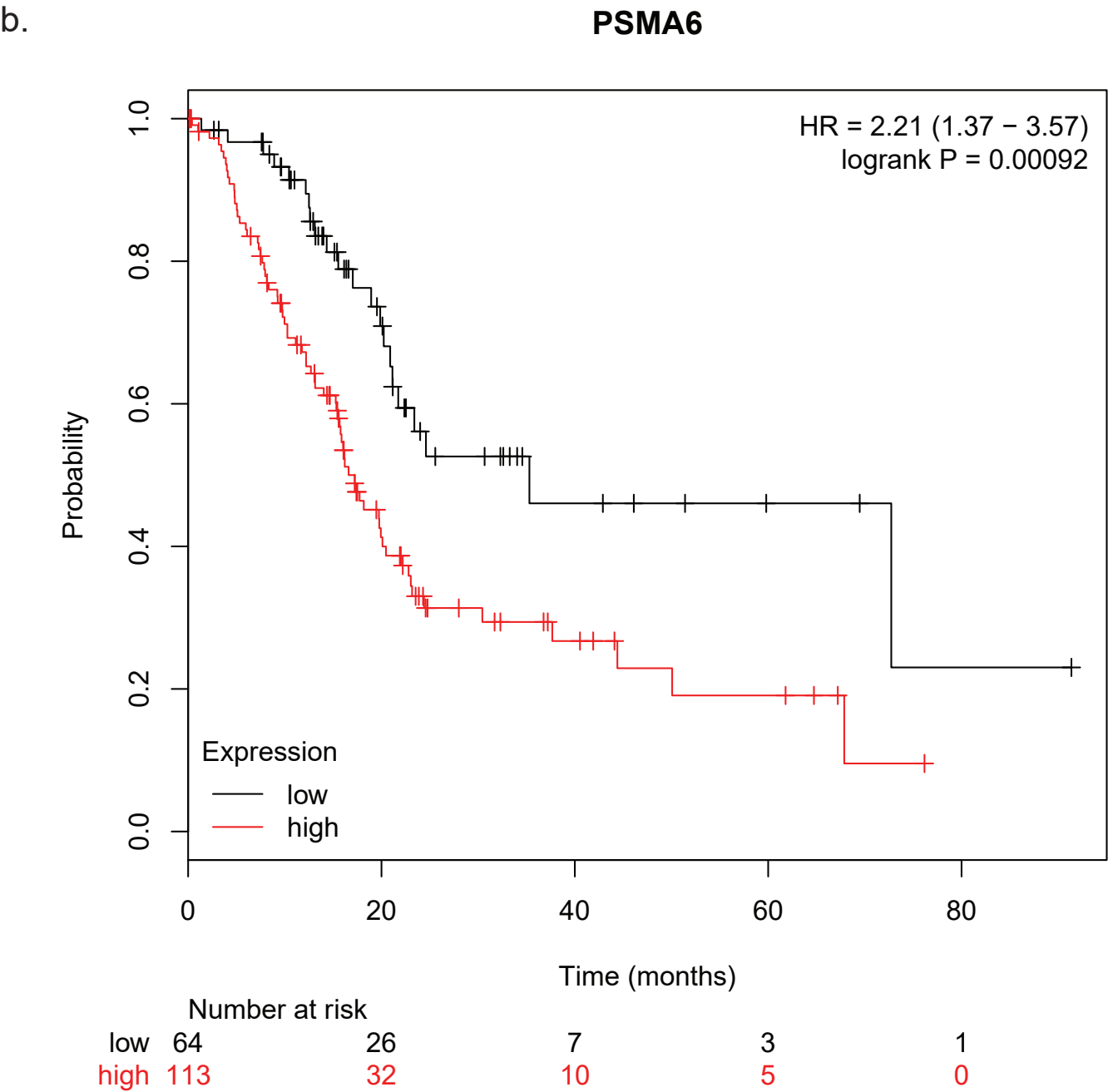

Supplement: Supplementary file 8 — a) PSMA6 expression query with cBioPortal tool from the TCGA Research Network. b) Kaplan-Meier plot of high and low PSMA6 expression in PDAC patient samples and overall survival. (PDF 29422 kb) [file 12885_2019_5455_MOESM8_ESM.pdf]

7-AAD vs Annexin V: Bortezomib

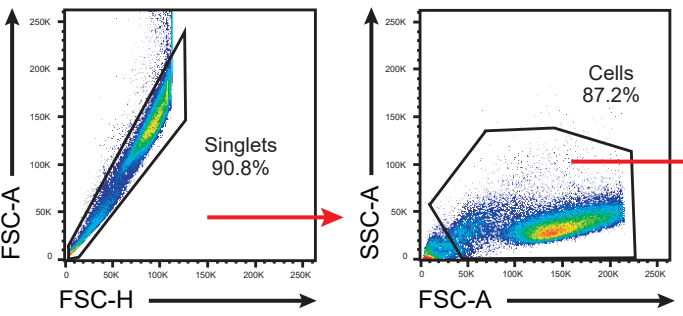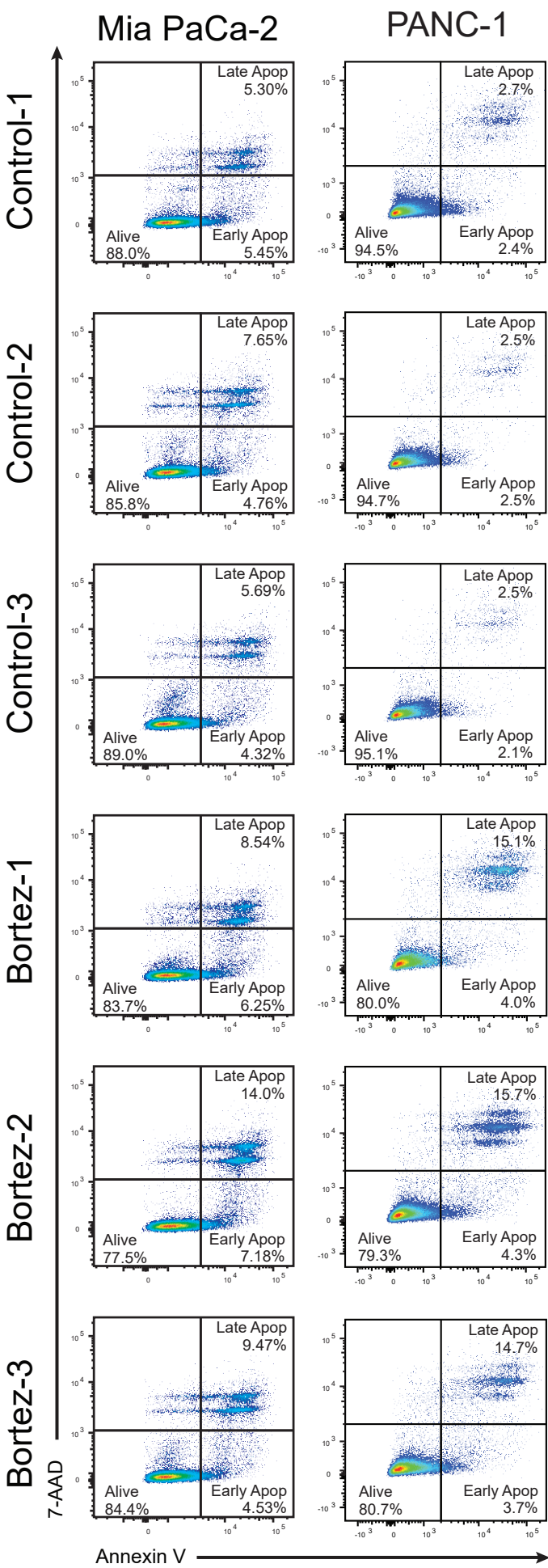

Supplement: Supplementary file 9 — Complete flow cytometry panel for 7-AAD and Annexin V staining in Mia PaCa-2 and PANC-1 cells after 48 h of treatment with 0.001 μM bortezomib or DMSO control (controls 1–3) (see Fig. 5b–d). (PDF 743 kb) [file 12885_2019_5455_MOESM9_ESM.pdf]
